# Is single-shot antibiotic prophylaxis really enough for standard OMF-surgeries?

**DOI:** 10.1007/s00784-026-06756-4

**Published:** 2026-02-02

**Authors:** Lara Schorn, Daman Deep Singh, Felicitas Mrochen, Melanie Kempe, Felix Schrader, Majeed Rana, Christoph Sproll, Julian Lommen, Insa Joost

**Affiliations:** 1 Department of Oral-Maxillofacial and Facial Plastic Surgery, University Hospital Düsseldorf, Moorenstr. 5, 40225 Düsseldorf, Germany; 2 Department of Pharmacy, Helios Clinic Schwelm, Dr.-Moeller-Str. 15, 58332 Schwelm, Germany; 3 Institute of Medical Microbiology and Hospital Hygiene, Heinrich Heine University, Universitätsstr. 1, 40225 Duesseldorf, Germany

**Keywords:** Antibiotic prophylaxis, OMF surgeries, Single-shot antibiotic, Prolonged antibiotic prophylaxis, Length of hospital stay

## Abstract

**Objectives:**

Antibiotic resistance and the development of multidrug-resistant pathogens are on the rise worldwide. This is mainly due to inappropriate or excessive use of antibiotics. There is a lack of evidence-based guidelines and studies for the administration of antibiotics in OMF surgery. In this study, a new shortened antibiotic prophylaxis regimen for six standard OMFS procedures (Fracture repair, plate removal, orthognathic surgery, TUROP, ND and bone augmentation) was introduced and evaluated for clinical outcomes and feasibility.

**Materials and methods:**

856 patients were enrolled in this study. They were monitored for postoperative complications such as dehiscence, fistula, signs of clinical infection, and laboratory markers of inflammation. Subgroup analyses were performed for clinically relevant procedure categories, including fractures, plate removal, and orthognathic surgery.

**Results:**

After risk adjustment, reduced-duration antibiotic prophylaxis was not associated with adverse postoperative outcomes. Furthermore, a more restrictive administration as a single shot was associated with a shorter length of hospital stay (from 5.83 (± 7.92) to 4.32 (± 4.88) days (p = > 0.001)).

**Conclusions:**

A single dose of antibiotics was found to be sufficient for the oral and maxillofacial procedures evaluated. Implementation of a new policy of reduced antibiotic prophylaxis coincided with a shorter length of hospital stay.

**Clinical relevance:**

This study supports the growing body of evidence suggesting that shorter, procedure-tailored antibiotic regimens can be safe and effective, potentially reducing antimicrobial resistance and adverse drug effects without compromising surgical outcomes.

## Introduction

The increasing incidence of multidrug-resistant pathogens such as methicillin-resistant Staphylococcus aureus (MRSA) and multidrug-resistant Gram-negative (MRGN) bacteria, as well as antimicrobial resistance in general, is placing a growing burden on prophylactic and therapeutic options in healthcare [1]. Although recent restrictive measures have slowed and, in some cases, reversed the increase, the problem of emerging antibiotic resistance remains a medical challenge [2, 3].

In oral and maxillofacial surgery, especially in intraoral procedures, the wound area is colonized by bacteria [4]. In addition, the anatomical conditions in the mandibular region are particularly conducive to bacterial colonization [5]. Favored by the highly colonized bacterial oral flora, a non-sterile surgical site, wound contamination and consequently wound infection are more likely to occur postoperatively. Possible causes include inadequate oral hygiene, fractured teeth, teeth in fracture gaps, infected cysts, pre-existing medical conditions that predispose to infection such as diabetes mellitus, former radiation or current immunosuppression [6]. These mixed bacterial infections consist of a predominantly anaerobic, mixed gram-positive and gram-negative environment [6].

In addition to appropriate wound care and procedures carried out under strict aseptic precautions, antibiotic prophylaxis provides an additional safeguard. It is especially important to prevent the wide range of potentially pathogenic microorganisms from the highly colonized environment of the mouth and throat from establishing infections, especially to the bone. For prophylaxis and therapy, aminopenicillins with ß-lactamase inhibitors are frequently used (e.g. Amoxicillin-clavulanic acid). The bactericidal combination of the two active components covers an extended spectrum. In addition to relevant gram-positive bacteria as staphylococci, streptococci and *E. faecalis*, it includes gram-negative bacilli like *Escherichia coli or Proteus*, but also *Haemophilus influenzae*, as well as the majority of the anaerob oral flora [7]. In case of penicillin allergy, lincosamide antibiotics, e.g. clindamycin, are often the drug of choice. It bacteriostatically inhibits the growth of *Staphylococcus sp.*, *Streptococcus pneumoniae* and *S.pyogenes*, anaerobes (such as *Fusobacterium sp.*, *Actinomyces sp.*, *Bacteroides sp.*, *Peptostreptococcus sp.* and *Peptococcus sp.*) [8, 9], . Alternatively, cephalosporins such as cefazolin or ceftriaxone i.v. can be used. While group 1 cefazolin is mainly effective against gram-positive pathogens, group 3 ceftriaxone is more effective against gram-negative pathogens [7].

Pre- and perioperative antibiotic prophylaxis plays a crucial role in minimizing the risk of post operative infections [4]. However, several studies have demonstrated the beneficial effects of more restrictive antibiotic use. Therefore, studies comparing selective and comprehensive peri- and postoperative antibiotic prophylaxis have been conducted in patients undergoing oral and maxillofacial surgery [10, 11]. It is hypothesized that reduced antibiotic administration could relieve inpatient capacity and accelerate patient discharge. Shorter hospital stays are also associated with a reduced risk of nosocomial infections [12, 13]. This risk reduction would be reflected in improved, i.e. reduced, morbidity and mortality [14].

This study evaluated postoperative complications in patients undergoing six standard OMFS procedures at an Oral and Maxillofacial Surgery department at a german tertiary care hospital while being treated with either only single-shot antibiotic prophylaxis or a prolonged scheme of postoperative prophylactic antibiotic use of $\:\ge\:$48 h. In addition, the duration of the inpatient stay and the total antibiotic consumption of the OMFS ward were examined. Based on the international literature, it was hypothesized that single-shot antibiotic prophylaxis would provide similar clinical outcomes compared to a prolonged regimen of postoperative prophylactic antibiotics.

## Materials and methods

In this observational study, data were retrospectively (01.01.2018 to 31.05.2019) and prospectively (01.06.2019 to 31.12.2020) collected from the University Hospital Düsseldorf (UKD) database. A randomization did not take place. To avoid duplicates, data were initially collected using patient number, name, and date of surgery. Later data were pseudonymized for analysis. Microsoft Excel for Mac 2020 (version 16.35; 20030802 and later) was used for data collection. Patients meeting the inclusion criteria who were registered in the UKD database between 01.01.2018 and 31.12.2020 were included in the study.

**Fig. 1:**
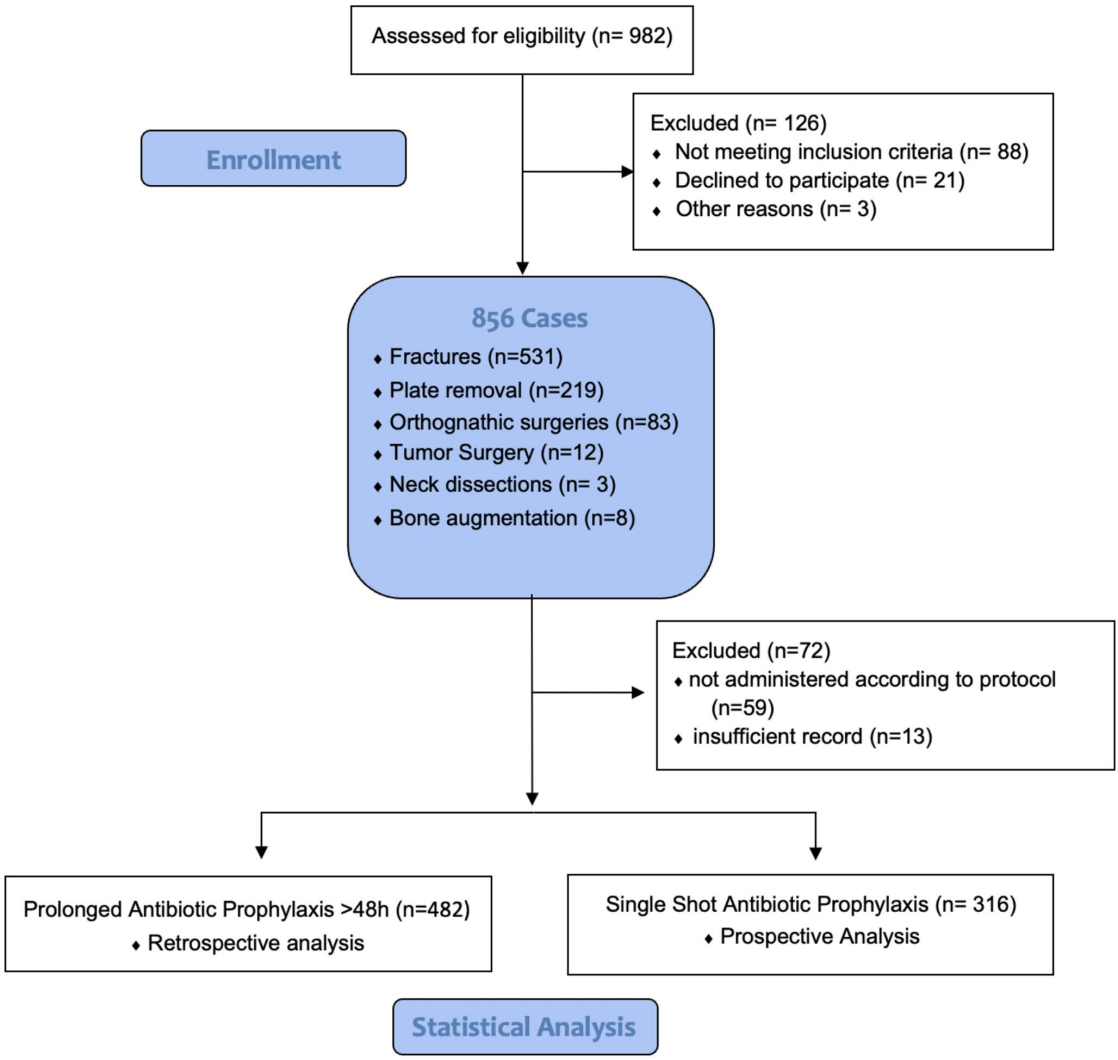
Patient flow diagram

From 01.01.2018 to 31.05.2019 a prolonged antibiotic scheme of 48 h (and longer) was applied to all included patients. On 01.06.2019 a current literature based internal guideline was introduced including only intraoperative single shot antibiotic coverage for the six surgeries involved. Therefore, from 01.06.2019 to 31.12.2020 only a single shot antibiotic regimen was applied (Fig. 1).

Flow diagram illustrating the progression of participants through the study, which includes both retrospective and prospective arms. The diagram outlines participant enrollment, allocation to study groups, follow-up, and final inclusion in the statistical analysis.

Inclusion criteria were undergoing of one of six standard OMF-Surgeries (open reduction and internal fixation with osteosynthesis plate of OMF-Fractures, removal of at least one osteosynthesis plate, orthognathic surgery – only bimaxillary procedures, intraoral ablative tumor surgery (exclusively intraoral tumors without further surgical steps such as plastic reconstruction or neck dissection), exclusively neck dissection and intraoral bone augmentation), age 18–90 years, the ability to read and understand German and informed consent. Exclusion criteria were lack of or inability of informed consent, patients with cocommittant infections that required antibiotic therapy, and patients not meeting inclusion criteria. In the plate removal subgroup, two procedures performed due to infection were excluded from the analysis, ensuring that only elective cases were considered.

Evaluated data included: type of operation, gender, age, BMI, allergies, general illnesses (divided into subcategories: neurological, pulmological, cardiovascular, infectiological, nephrological, endocrinological, cancerous, psychological and other), medication, type of antibiotic prophylaxis (single-shot vs.$\:\ge\:$48h), type of agent used, form of administration (oral or intravenous), clinical signs of infection (calor, rubor, dolor, tumor, pus), wound dehiscence (mechanical = separation of the wound edges in the absence of infection, caused by physical or technical factors that compromise wound closure, and infectious = separation of the wound edges secondary to an infectious process in the surgical site), surgery duration, surgical approach, laboratory results of parameters indicating infection (C-reactive protein, white blood cell count (WBC)), inpatient length of stay, postoperative complications (wound dehiscence, abscess, infection) and possible revision surgery.

### Surgeries and standard of care

All orthognathic procedures were performed following proper orthodontic preparation. During the observation period, surgeries were carried out by always the same three surgeons applying almost identical surgical techniques. Maxillary movements were performed using a Le Fort I osteotomy in all included patients. In the mandible, bilateral sagittal split osteotomy (BSSO) was performed according to Obwegeser and subsequent modifications. For the other types of surgery (e.g., trauma, augmentation, plate removals), different surgeons were involved. Nevertheless, all procedures were consistently performed under the supervision of, or directly by, board-certified oral and maxillofacial surgeons, thereby ensuring a uniform specialist standard of care throughout the entire study period. Throughout the study period, the responsible surgical team remained unchanged. No relevant changes occurred with regard to surgical technique, suture material, or planning protocols. Average operating times were comparable across the study period. The interval between trauma and surgical treatment varied by fracture type: mandibular fractures were generally treated within 0–2 days, whereas midfacial fractures were managed after 4–9 days. In cases of open mandibular fractures, preoperative antibiotics were administered (e.g. Aminopenicillins with ß-lactamase inhibitors 1-1-1 until surgery). In all cases, trauma or orthognathic, internal fixation was achieved using titanium miniplates. In fractures the method of osteosynthesis was consistently open reduction and internal fixation (ORIF) using titanium plates. For surgeries longer than 3 h, a second dose of antibiotics was administered. Trauma patients typically remained hospitalized for 2–4 days postoperatively. Patients with mandibular fractures are admitted immediately after trauma and operated on without delay, whereas patients with midfacial fractures are admitted depending on severity. At the time of the study, patients undergoing plate removal were routinely hospitalized for 2 days. Orthognathic surgery patients routinely remained hospitalized for 5–6 days postoperatively. Postoperative radiographic controls were routinely performed and reviewed to confirm adequate reduction. There was no standardized scientific follow-up protocol; instead, patients received regular postoperative surgical follow-up based on clinical necessity. 

### Antibiotic consumption

Intravenous antibiotic consumption was calculated as defined daily doses (DDD) per 100 patient days, in accordance with the WHO ATC/DDD methodology. Data collection was limited to systemic antibiotics administered intravenously. Consumption data were extracted via the AVS (Antibiotikaverbrauchssurveillance) module provided by the Robert Koch Institute (RKI), using routine hospital pharmacy dispensing data. Patient-day denominators were obtained from institutional administrative records.

### Statistical analysis

Statistical analysis was performed by an independent professional statistical institute (punkt05 Statistik, Düsseldorf, Germany). All analyses were conducted according to established standards for biomedical research, including systematic testing for potential confounders, appropriate adjustment methods, and verification of model assumptions. A required minimum sample size of *n* = 305 patients per group was previously calculated at a significance level of *p* < 0.05 (for ANOVA (fixed effects, omibus, one-way) - effect size 0.5 with 2 unrelated study groups, G*Power, Heinrich-Heine University Düsseldorf) [15]. IBM SPSS statistics for Mac version 26 (SPSS Inc., Chicago, IL, USA) was used for data analysis. Means and standard deviations were evaluated (mean ± SD). The chi-square test, Likelihood-Ratio and Cramer’s V were calculated for normally distributed data. The Mann-Whitney U test and the Wilcoxon test were used for non-normally distributed data. For fractures, removals of osteosynthesis plates, and the total collective, a sufficiently large collective was available to determine asymptotic significance. Clinical signs of postoperative infection (yes/no) was defined as the primary outcome and analyzed using multivariable logistic regression. The antibiotic prophylaxis regimen (single-shot vs. >48 h) was included as the main exposure variable. Models were adjusted a priori for age, sex, body mass index, comorbidity burden, procedure category and fracture type. Postoperative inflammatory markers, including C-reactive protein levels and leukocyte counts, as well as length of hospital stay (in days), were evaluated as secondary outcomes using multivariable linear regression models. All secondary outcome models included the antibiotic prophylaxis regimen as the exposure of interest and were adjusted for the same set of covariates. Comorbidity burden was defined as the number of documented pre-existing conditions per patient. Adjusted odds ratios (ORs) with 95% confidence intervals (CIs) were calculated. Due to low event numbers, fracture types with insufficient postoperative infections were excluded from multivariable modeling. Additional stratified analyses were performed for fracture types with sufficient event counts. A two-sided p value < 0.05 was considered statistically significant. Subgroup analyses were predefined to explore potential differences between clinically relevant procedure categories and were restricted to groups with sufficient sample size and event counts to ensure model stability. Surgical procedures were categorized into fractures, plate removals, orthognathic surgery, neck dissection, augmentation procedures, and tumor surgery. Due to the small number of tumor surgeries and neck dissections, these procedures were excluded from subgroup-specific inferential analyses and are reported descriptively only. Inferential analyses focused on fractures, plate removals, and orthognathic surgery, which represented the majority of cases and provided sufficient event numbers for multivariable modeling. For orthognathic surgeries, augmentations, and tumor resections, a continuity correction was additionally applied due to frequencies < 5 and the Fisher-Yates-Test was determined.

## Results

### Characterization of the cohort

During the complete study period from 01.01.2018 to 31.12.2020, in total 856 patients met the inclusion criteria. 859 cases were evaluated. The variation in sample size is explained by the fact that patients who were operated more than once were counted once within a case group. Of these 856 cases, the following surgical procedures were performed: A total of 531 fractures were included in the analysis. Midfacial fractures comprised Le Fort I (*n* = 10, 1.9%), Le Fort II (*n* = 20, 3.8%), Le Fort III (*n* = 7, 1.3%), orbital floor fractures (*n* = 87, 16.4%), zygomatic bone fractures (*n* = 148, 27.9%), alveolar buttress fractures (*n* = 1, 0.2%), anterior wall of the maxillary sinus (*n* = 2, 0.4%), and isolated zygomatic arch fractures (*n* = 82, 15.4%). Mandibular fractures accounted for 174 cases (32.8%), 219 osteosynthesis plate removals, 83 orthognathic surgeries, 12 ablative tumor surgeries, 3 neck dissections and 8 intraoral bone augmentation cases and were included in this study. 482 (56.6%) patients received prolonged antibiotic prophylaxis of $\:\ge\:$48 h. 316 (37.1%) patients received only a single dose of antibiotic prophylaxis. In 72 (8.28%) cases, antibiotic prophylaxis was either not administered according to protocol or not recorded, resulting in exclusion from the study.

Sociodemographic data revealed an average age of 41.9 years, including 29.7% (*n* = 259) women and 70.2% (*n* = 611) men. The mean body mass index (BMI) was 24.3. Underlying diseases included nephrological (1.7%), neurological (4.1%), cardio-vascular (17.4), pulmonary (9.2%) infectious (4.8%), endocrinological (9.8%), oncological (7.8%), psychiatric (5.8%), and other diseases (4.3%), as well as previous or current infection with Methicillin-resistant Staphylococcus aureus (MRSA) or multi-resistant gram-negative bacteria (3/4-MRGN) (1.5%). 

### Post-operative complications

**Fig. 2:**
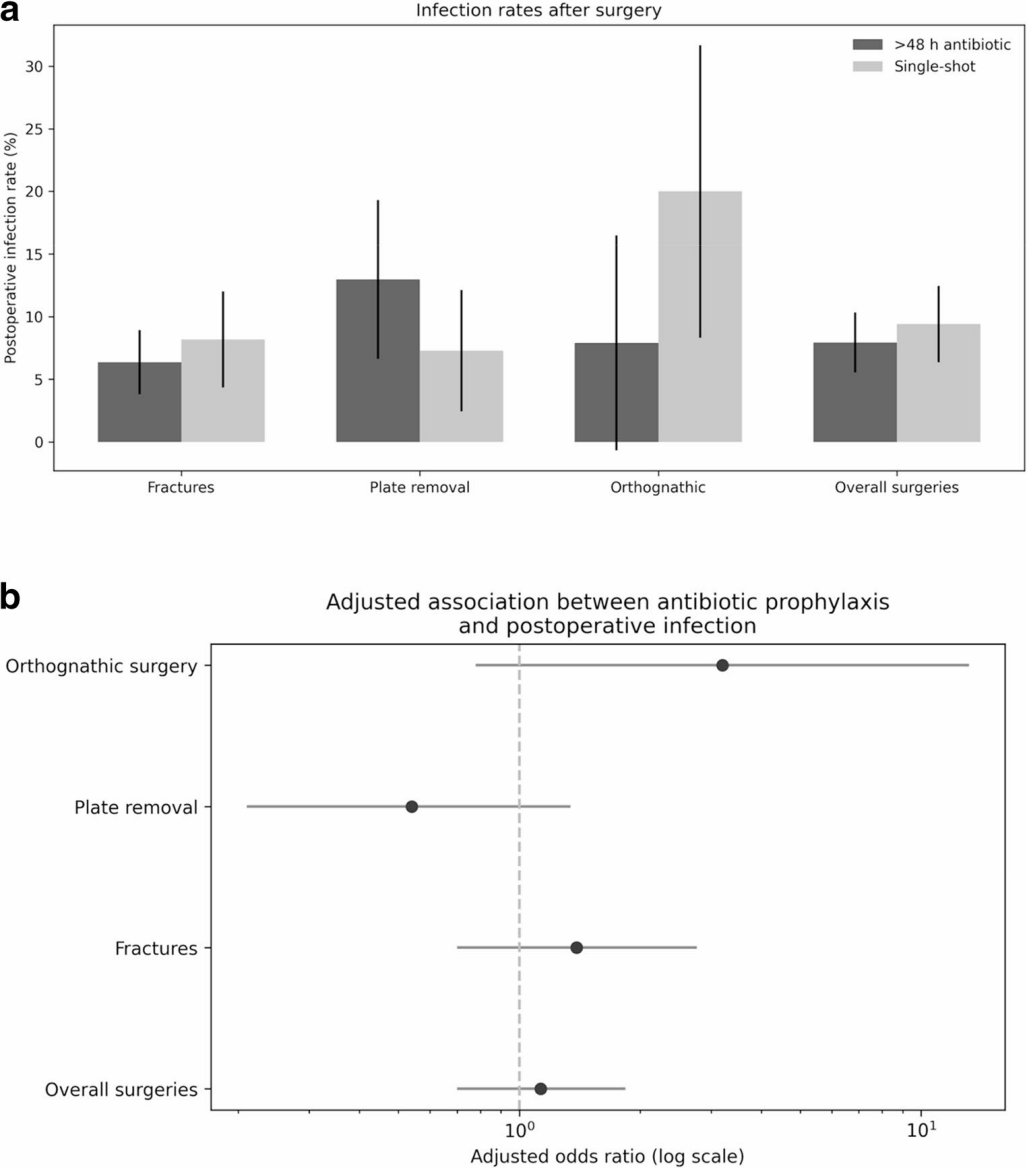
**a**. Clinical signs of infection by surgical procedure and antibiotic prophylaxis. Unadjusted postoperative infection rates with 95% confidence intervals for fractures, plate removal, orthognathic surgery, and overall surgeries, comparing single-shot antibiotic prophylaxis with prophylaxis administered for more than 48 hours. The figure is intended for descriptive visualization; adjusted associations are reported in the multivariable analyses. **b**. Multivariable analysis of postoperative infection. Forest plot showing adjusted odds ratios with 95% confidence intervals for postoperative infection comparing single-shot antibiotic prophylaxis with prophylaxis administered for more than 48 hours across procedure subgroups. Odds ratios were obtained from multivariable logistic regression models adjusted for age, sex, body mass index, comorbidity burden, and procedure category, as applicable. Subgroup analyses were exploratory and not powered to detect small differences. Odds ratios crossing unity indicate no statistically significant association

Due to the small number of tumor surgeries, neck dissections and bone augmentations, further statistical analysis was performed only for fracture treatment, plate removal, orthognathic surgeries, and overall surgeries. In unadjusted subgroup analyses, postoperative infection rates were 6.36% versus 8.16% for fractures (*p* = 0.48), 12.96% versus 7.27% for plate removal (*p* = 0.18), and 7.89% versus 20.00% for orthognathic surgery (*p* = 0.21) when comparing prophylaxis > 48 h with single-shot prophylaxis. Overall infection rates were 7.93% versus 9.40%, respectively (*p* = 0.46; Fig. 2a). For Fractures in the multivariable logistic regression analysis, single-shot antibiotic prophylaxis was not independently associated with the occurrence of postoperative infection compared with prophylaxis administered for more than 48 h (adjusted OR 1.45, 95% CI 0.72–2.94; *p* = 0.298). Age, sex, body mass index, comorbidity burden, and fracture type were not significantly associated with postoperative infection in the adjusted model. Stratified multivariable analyses were conducted for different fracture types with sufficient numbers of postoperative infections (Mandibular fractures, zygomatic bone fractures, Orbital fractures and Le-Fort fractures). In patients with mandibular fractures, the antibiotic prophylaxis regimen was not independently associated with postoperative infection after adjustment for age, sex, body mass index, comorbidity burden, and fracture characteristics (adjusted OR 1.90, 95% CI 0.72–4.98; *p* = 0.192). In patients with zygomatic-orbital fractures, no significant association between antibiotic regimen and postoperative infection was observed (adjusted OR 0.66, 95% CI 0.07–6.40; *p* = 0.717).

For other fracture types, multivariable modeling was not performed due to insufficient event numbers.

In the orthognathic surgery subgroup, the association observed in unadjusted analyses did not persist after multivariable adjustment (adjusted OR 3.20, 95% CI 0.78–13.15; *p* = 0.106). The wide confidence interval reflects limited statistical power due to the small number of events.Although the point estimate suggested a higher odds of infection with single-shot prophylaxis, the confidence interval was wide, indicating substantial uncertainty due to limited event numbers.

Overall surgeries, in the multivariable logistic regression model, single-shot prophylaxis was not independently associated with postoperative infection compared with prophylaxis > 48 h (adjusted OR 1.13, 95% CI 0.70–1.84; *p* = 0.616), adjusted for age, sex, BMI, comorbidity burden and procedure category (Fig. 2b).

There was no significant difference in laboratory parameters indicative of infection (CRP and white blood cell count) for orthognathic surgeries while there was a significant difference in CRP values for removal of osteosynthesis plates (> 48 h antibiotic prophylaxis: 1.74 $\:\pm\:$2.50 mg/dl versus single-shot 2.69 $\:\pm\:$3.14 mg/dl, *p* = 0.046, Fig. 4a + b). Overall surgeries, in multivariable linear regression models adjusting for age, sex, BMI, and comorbidity burden, and surgical procedure, single-shot prophylaxis was not associated with higher postoperative CRP (β = 0.33 mg/dl, 95% CI −0.38 to 1.03; *p* = 0.365) or postoperative leukocyte counts (β = 0.32 mg/dl, 95% CI −0.54 to 1.17; *p* = 0.468) compared with prophylaxis >48 h.

**Fig. 3:**
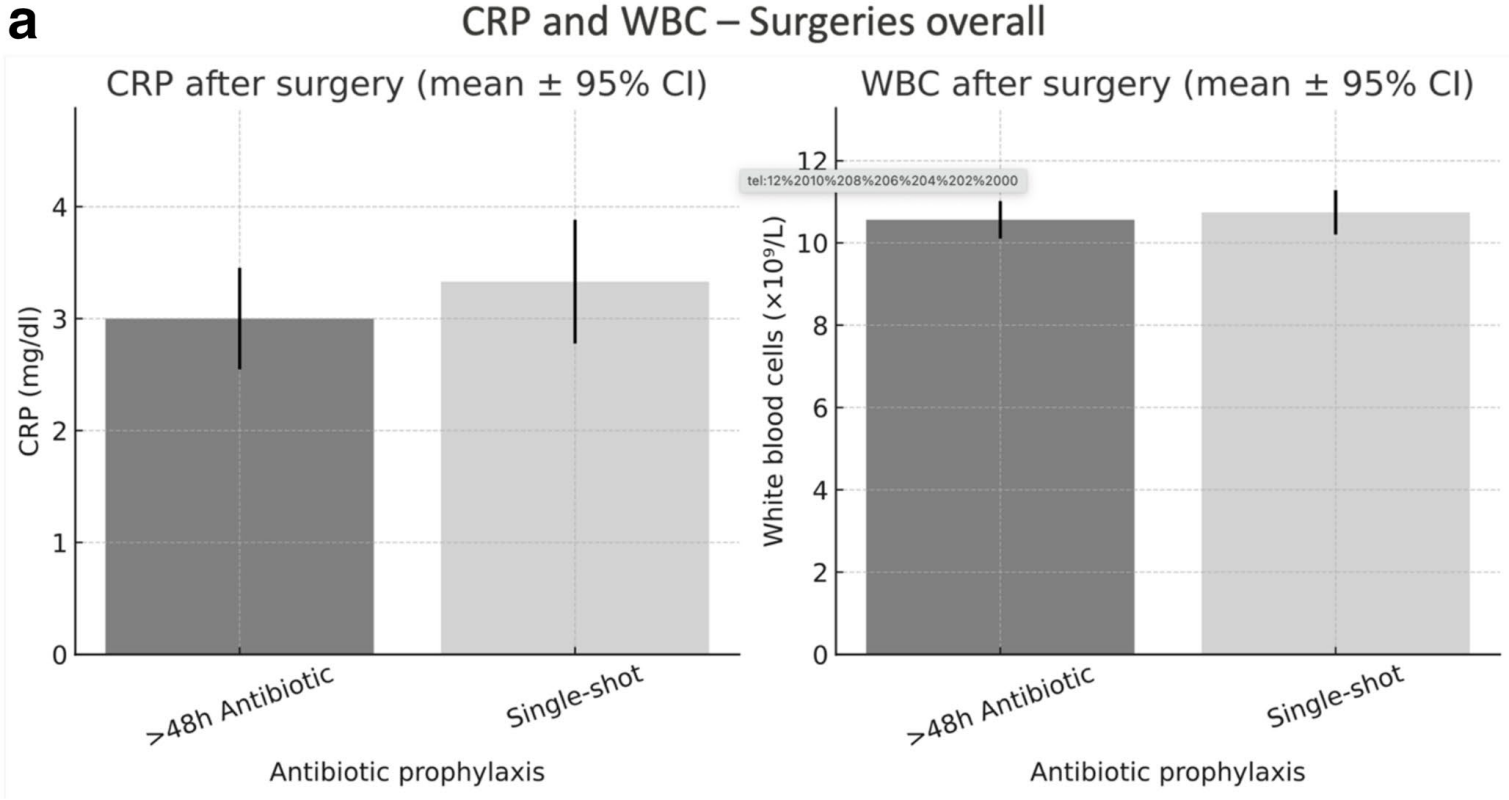

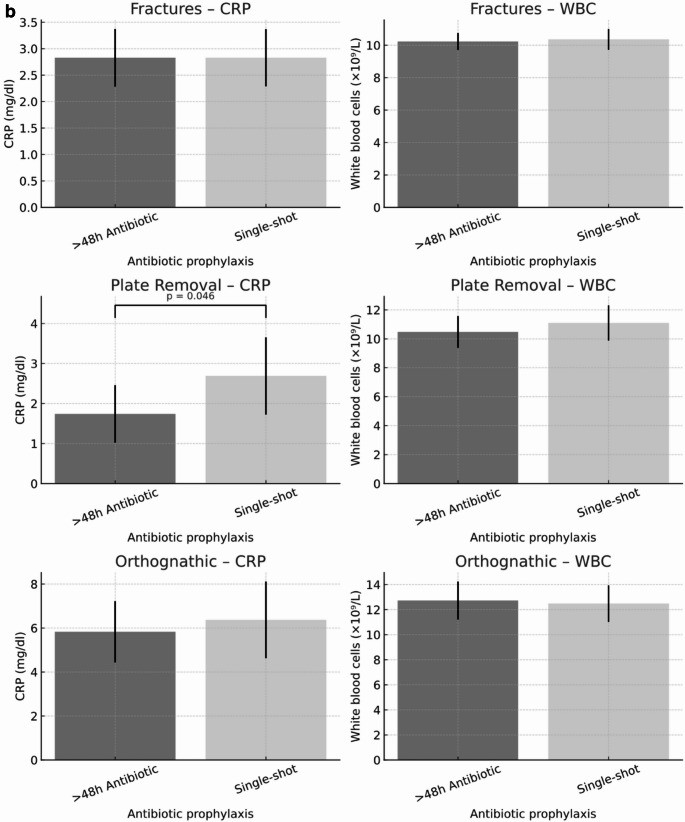
**a**. Postoperative inflammatory markers for all surgeries. Postoperative inflammatory markers in patients receiving either single-shot or prolonged (> 48 h) antibiotic prophylaxis. Bars represent mean values with 95% confidence intervals. (A) C-reactive protein (CRP, mg/dl). (B) White blood cell count (WBC, ×10⁹/L). No significant differences were observed between antibiotic regimens for either marker. The figure is intended for descriptive visualization; unadjusted comparisons are shown, and adjusted associations are reported in the multivariable analyses. **b**. Postoperative inflammatory markers by surgical procedure and antibiotic prophylaxis. Postoperative C-reactive protein (CRP, mg/dl) and white blood cell (WBC, ×10⁹/L) levels in patients receiving either single-shot or prolonged (>48 h) antibiotic prophylaxis, stratified by surgical procedure (fracture treatment, osteosynthesis plate removal, and orthognathic surgery). Bars indicate mean values with 95% confidence intervals. CRP levels after plate removal were significantly higher in the single-shot group compared to the prolonged prophylaxis group (p = 0.046). The figure is intended for descriptive visualization; unadjusted comparisons are shown, and adjusted associations are reported in the multivariable analyses

All other types of surgeries showed no significant differences in terms of clinical infection, wound dehiscence, or laboratory parameters indicative of infection (Figs. 2a and 3a+b). 

### Length of stay

**Fig. 4:**
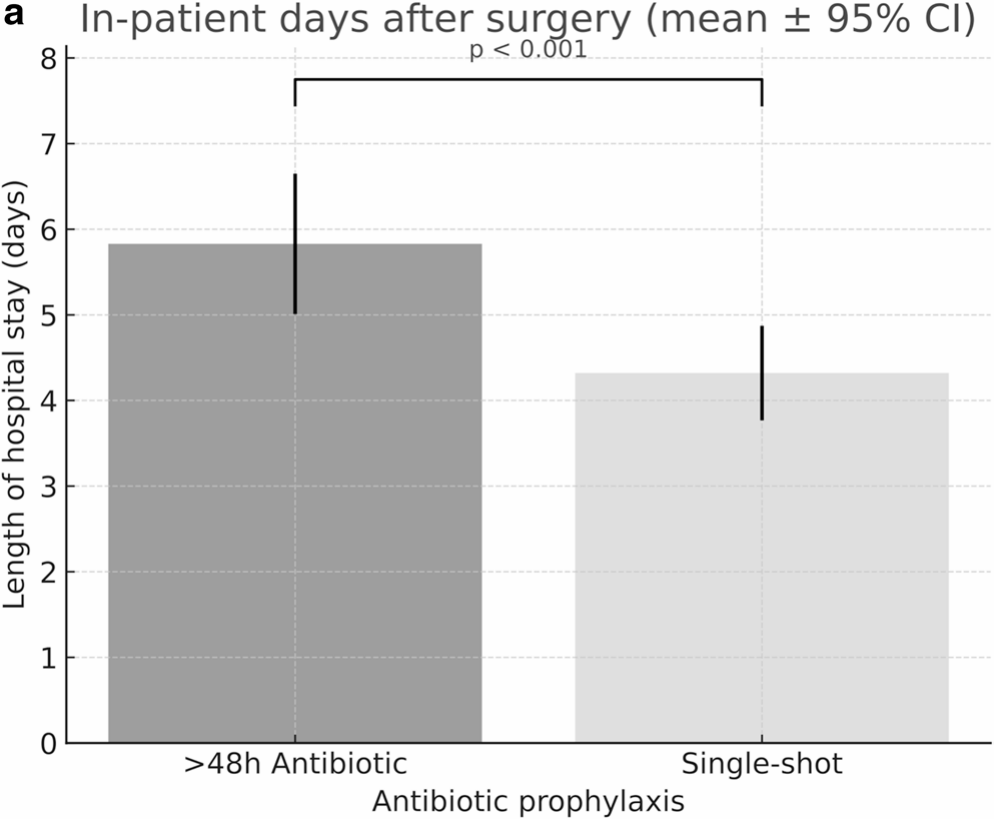

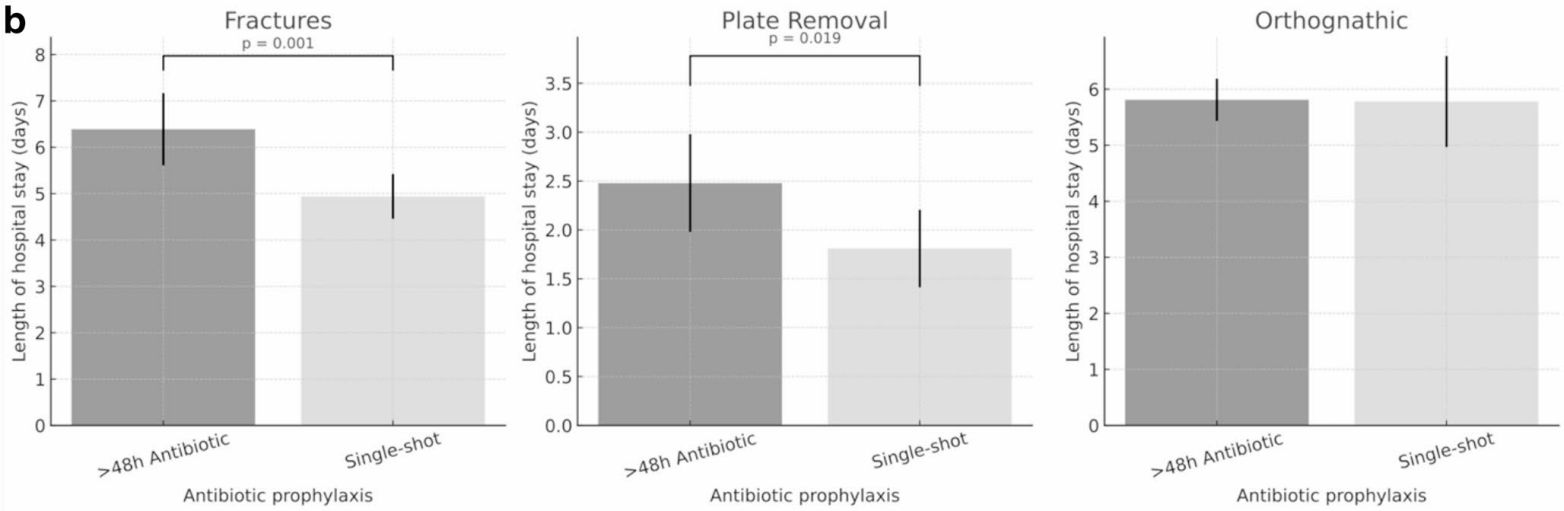
**a**. Length of in-patient stay by antibiotic prophylaxis. Length of hospital stay in patients receiving either single-shot or prolonged (>48 h) antibiotic prophylaxis. Bars represent mean values with 95% confidence intervals. A significantly shorter hospital stay was observed in the single-shot group compared with the prolonged prophylaxis group (p < 0.001). Differences should be interpreted as associations rather than causal effects. **b**. Length of in-patient stay by antibiotic prophylaxis and procedure. Length of hospital stay in patients receiving either single-shot or prolonged (>48 h) antibiotic prophylaxis, stratified by surgical procedure (fracture treatment, osteosynthesis plate removal, and orthognathic surgery). Bars represent mean values with 95% confidence intervals. A significantly shorter hospital stay was observed in the single-shot group for fracture treatment (p = 0.001) and plate removal (p = 0.019), while no significant difference was found in orthognathic surgery. Differences should be interpreted as associations rather than causal effects

A significant difference depending on the length of antibiotic prophylaxis could also be demonstrated for the overall in-patient length of stay (in days, Fig. 4a). In a multivariable linear regression model adjusted for age, sex, BMI, comorbidity burden and procedure category, single-shot prophylaxis was associated with a shorter length of hospital stay compared with prophylaxis >48 hours ((>48h antibiotic prophylaxis: 5.83 ±7.92 days versus single-shot 4.32 ±4.88 days, 95% CI −2.45 to −0.74; p < 0.001 ). Significant differences in the duration of hospital stay for the surgery subgroups of fracture osteosyntheses (>48h antibiotic prophylaxis: 6.39 ±5.74 days versus single-shot 4.94 ±2.89 days, p=0.001) and plate removal (>48h antibiotic prophylaxis: 2.48 ±2.60days versus single-shot 1.81 ±2.08 days, p=0.019) (Fig. 4b).

### Antibiotic consumption

 Annual inpatient antibiotic consumption data from the OMFS clinic showed a decrease from 63.8 defined daily doses (DDD)/100 patient days in 2018 to 47.0 DDD/100 patient days in 2020.

## Discussion

Overall results suggest that patients treated under a more restrictive antibiotic prophylaxis regimen did not experience worse postoperative outcomes. The length of stay for patients undergoing fracture treatment and removal of osteosynthesis plates was reduced without increasing post-operative complications. The present study focused on procedure categories with sufficient case numbers to allow meaningful statistical analysis. Tumor surgeries and neck dissections were intentionally excluded from inferential subgroup analyses due to the very small number of cases, as inclusion of these procedures could lead to misleading conclusions regarding soft-tissue surgery.

In recent years, an increasing number of studies have addressed the need for and duration of perioperative antibiotic prophylaxis in oral and maxillofacial surgery. To date, no differences have been demonstrated between short (≤ 24 h) and prolonged (≥ 72 h) perioperative prophylaxis [11]. Even when comparing a 24-hour prophylaxis regimen to one lasting five days, extended administration of antibiotics failed to show additional benefit. A single dose has proven sufficient for prophylaxis [16] and this is in accordance with recently published german guidelines (S3-Leitlinie: Klassen-Upgrade Perioperative und Periinterventionelle Antibiotikaprophylaxe Perioperative and Periinterventional Antibiotic Prophylaxis in Surgery Langversion 5.0 - September 2024, AWMF-Registernummer: 067 − 009.

Fördernummer beim Gemeinsamen Bundesausschuss (G-BA): 01VSF21022) [17]. Overall, the results of our study are consistent with these findings. Interestingly, the duration of hospital stay showed a significant correlation. Overall, the duration of stay dropped from 5.83 to 4.32 days in the group of patients receiving a single-shot perioperative antibiotic. Shortening the length of stay leads to a reduction in the risk of nosocomial infections, with lower nursing costs, overall costs and increased patient turnover. Every day less spent in hospital could potentially reduce the burden on a fragile hospital system, benefiting patients, hospitals and health insurers alike.

In terms of operative complications, in the largest subgroup studied, the maxillofacial trauma cases and consequent plate removal, no significant difference in postoperative outcomes due to the different durations of antibiotic administration was seen. In fracture-specific analyses, no statistically significant association between antibiotic prophylaxis regimen and postoperative infection was observed for any fracture type. While point estimates differed between subgroups, confidence intervals were wide and overlapped substantially, reflecting limited event numbers within individual fracture categories. These findings suggest that there is no clear evidence supporting a reduction in postoperative infection risk with prolonged antibiotic prophylaxis in specific fracture types; however, the results should be interpreted with caution due to limited statistical power. This equals other international studies. Blatt et al. state that mandibular fractures as well as Le-Fort 1 and 2 fractures benefit from perioperative prophylaxis for up to 24 h. There is no further reduction in the risk of infection with longer administration [18]. Even open fractures in the orthopedic area (the facial area tends to be less prone to infection because of its rich blood supply) have not been shown to benefit from prophylactic antibiotics for more than 24 h postoperatively [19]. However, due to intraoral bacterial colonization and possibly teeth in the fracture line, mandibular fractures should not be equated with other open fractures, as these are often contaminated. An open mandibular fracture is associated with a higher risk of infection than a closed mandibular fracture [20]. Whether this justifies extended antibiotic prophylaxis is controversially discussed in literature. Some studies show the superiority of antibiotic coverage in open mandible fractures compared with omission of antibiotic prophylaxis [21, 22]. Other studies found no benefit of antibiotic prophylaxis over 24 h or generally postoperatively in open and closed mandibular fractures [23–25]. In our data, for mandibular fractures, which represented the largest fracture subgroup, multivariable analysis did not demonstrate an independent association between prolonged antibiotic prophylaxis and postoperative infection. Although the adjusted point estimate suggested a higher odds ratio for single-shot prophylaxis, this effect did not reach statistical significance and was accompanied by a wide confidence interval, indicating uncertainty around the true effect size. In oral and maxillofacial surgery, the surgical approach can also be decisive for the choice of prophylaxis. If a transcutaneous approach were chosen for surgery on the zygomatic bone instead of an intraoral approach, the surgical site would be “clean” instead of “clean and contaminated”. Consequently, provided there are no other contraindications, it would be possible to operate without antibiotic coverage [26]. This usually does not comply with the aesthetic and minimally invasive principles of facial plastic surgery, but given the extraordinary facial blood supply and the possibility of an extraoral approach, one might argue that in some cases, even a single-shot antibiotic prophylaxis might be overtreatment. A study by Fay et al. evaluated differences in wound infections according to the duration of antibiotic prophylaxis in orbital fractures in a group of 1225 patients. In patients who did not receive antibiotic prophylaxis, there was no increase in the already low incidence of wound infection compared with patients who received a single course of antibiotic prophylaxis. However, patients receiving oral antibiotics for one week postoperatively, 12% experienced antibiotic-related adverse events [27]. In a large systematic review and metaanalysis, 9 studies reporting adverse events including 920 patients were analyzed. A higher risk for adverse events, mainly diarrhea, nausea and rash, was shown for patients receiving prolonged antibiotic prophylaxis compared to short courses (RR, 2.40; 95% CI, 1.20–3.54) [11]. Taken together, the fracture-specific analyses do not provide evidence for a clinically meaningful benefit of extending antibiotic prophylaxis beyond a single perioperative dose. Importantly, the findings of the present study should not be extrapolated to open mandibular fractures or other high-risk trauma scenarios involving extensive intraoral exposure, as these conditions are associated with substantially different contamination risks and were not specifically represented by distinct, reliably classifiable subgroups in our dataset.

In the subgroup of radical tumor operations, our cohort included only 12 cases which was unfortunately too small to create representative results. Blatt et al. [18] presented the benefits of perioperative antibiotic prophylaxis in oncological head and neck cases in a review in 2019. He differentiated between oncological neck surgery in “clean-contaminated” and “contaminated” surgical areas. A prophylaxis benefit was described for “clean-contaminated” wound regions. This is congruent with the results of a meta-study by Ariyan et al. from 2015 [28]. In a 2017 study however, Bartella et al. [29]. showed a reduction in surgical wound infections in tumor surgery in the neck and throat area with postoperative antibiotic prophylaxis for 5 days compared to perioperative prophylaxis alone as well as perioperative prophylaxis and postoperative antiseptic measures. Whether extended antibiotic usage is beneficial for patients undergoing oncological operations remains unclear. Generally, when choosing the optimal antibiotic regimen, the degree of contamination of the wounds must be taken into account as well as host factors, the duration of the surgical procedure and the extent of wound areas.

In the present study, unadjusted analyses suggested higher postoperative infection rates and wound dehiscence following single-shot antibiotic prophylaxis in orthognathic surgery. However, this association did not persist after adjustment for relevant patient-related confounders in multivariable analysis. The attenuation of the observed effect after adjustment indicates that the unadjusted difference was at least partly explained by confounding rather than by the duration of antibiotic prophylaxis itself. Orthognathic surgeries are per se planned procedures on comparatively young and healthy patients. At 2–6 h, the duration of surgery is also not unusually long for maxillofacial surgeries [30]. For surgeries longer than 3 h, a second dose of antibiotics is usually required, even with a “single shot” regimen. When a second dose of antibiotics was required, it was documented and administered by the anesthesia department. A study on surgical wound infections published in 2009 describes a statistical correlation between the duration of orthognathic surgery and the antibiotic substance administered and the incidence of postoperative infections [31]. Naros et al. [32] reported surgical site infections in 12.4% of orthognathic cases when intraoperative single-dose antibiotic prophylaxis was administered. According to Van Camp et al. [33], a generally increased risk of infection in patients with bilateral sagittal split osteotomy (BSSO) can be assumed. This seems plausible, as there naturally is a large exposed bone surface with temporary exposure to the oral cavity. In our study the type and distance of displacement was not evaluated. However, Naros et al. [32] found out, that there was also no correlation with many other co-factors such as duration of surgery and displacement distance or type of displacement. Studies have extensively researched possible sources of risk in conversion osteotomies. They all emphasize that even small errors in planning and execution can have a significant effect on the outcome. Starting with comprehensive planning, through correct surgical execution to appropriate aftercare. Morris et al. [34] and Patel et al. [35] agree on the necessity of clean surgical execution to reduce the risk of postoperative complications, including revision surgery and treatment. In our department, as a standard, the final conversion splint is ligated into the multiband apparatus and left in the oral cavity for 21 days post-surgery. The splint itself therefore might create a bacterial reservoir due to biofilm formation. Together with postoperatively contaminated wound conditions due to oral food intake, for example, it might favor infectious processes. According to Frieri et al. [36] these kinds of biofilms are a complicating factor in the containment of inflammation. Insufficient splint hygiene could be a relevant contributor to postoperative infection risk in orthognathic patients. Providing better cleaning instructions and clearer communication about this risk factor to patients could help to prevent infection and as a consequence prevent extending the duration of antibiotic prophylaxis. Taking these circumstances into account, surgeons may favor a prolonged use of antibiotic prophylaxis in orthognathic surgery cases, especially if foreign material such as the final conversion splint is left intraorally. Larger prospective studies are necessary to clarify the role of prolonged antibiotic administration in preventing post-operative complications in these types of surgeries, especially by differentiating between infectious and non-infectious complications. Obviously, meticulous post-operative oral hygiene is also recommended.

This study has several limitations. The study design should have been fully prospective and with a randomized study population in order to properly compare results. Due to a complete change in antibiotic management throughout the department, it was not possible to restart or continue prolonged antibiotic prophylaxis for study purposes only. Furthermore, study parameters should have been assessed with more detail. For example, an explicit distinction between infection and wound dehiscence, in particular between mechanical and infectious origin of the wound dehiscence could have provided more clarity about the severity of the course. Dehiscence, in particular, can be caused by a variety of different etiologies, and their severity and significance for the course of treatment and healing can vary considerably. A distinction should also have been made between early complications (up to 30 days after treatment) and late complications, as the underlying cause may be very different [37]. Unfortunately, there was often insufficient documentation to describe these complications in detail. Antibiotic-related adverse events such as diarrhea, gastritis, nausea, or bloating could not be systematically assessed in this retrospective study. These symptoms are often nonspecific in the postoperative setting and may also be influenced by dietary restrictions (e.g., soft diet, reduced oral intake) or other perioperative factors, making it difficult to attribute them directly to antibiotic prophylaxis. This represents a limitation, as potential side effects could not be adequately quantified to further support the rationale for reduced prophylaxis. It would also have been possible to distinguish between different fractures and the degree of contamination of the surgical approach (i.e. intraoral vs. extraoral approach). Due to the partially retrospective nature of data collection, information on smoking status and alcohol abuse was not consistently available and could therefore not be included in the multivariate analysis, although both are known to influence wound healing [38, 39]. However, the study population was not large enough to allow further differentiation of complications, approaches, and fractures. The interval between trauma and surgery differed depending on fracture type, with earlier intervention in mandibular fractures (0–2 days) and longer delays in zygomatic fractures (5–9 days). This variability, together with the administration of preoperative antibiotics in open mandibular fractures, may have influenced postoperative infection risk and should be considered when interpreting the results. Patients with documented antibiotic allergies were not excluded a priori. In such cases, alternative antibiotic regimens were prescribed according to clinical standards. Given the very small number of affected patients, this is unlikely to have influenced the overall findings, but it may limit comparability across all cases. Due to the different surgical indications and clinical presentations of the patients included, the overall study population was very heterogeneous. Further studies including more patients are warranted. In the subgroups, however, the patient population was comparable with typical patient populations for the respective disease. For example, a facial trauma study from the university clinic of Erlangen, Germany showed with 65.67% male and 34.33% female patients and an average age of 54.55 years a similar trauma patient population to ours [40]. According to the data collected by the Centre for Cancer Registry Data from 2019, tumors of the oral cavity and pharynx appear at an older age. Women are affected 2–3 years later than men, who in Germany are diagnosed at an average age of 64, and women at 66 [41, 42]. In comparison, our tumor patient population showed a slight upward shift in age. The heterogeneity of the overall study group was in this case not seen as a disadvantage. Rather, it reflects a realistic patient population in a maximum care hospital. As the groups were compared over the course of several years in the same hospital, it can be assumed that there is a similar distribution of the statistical parameters in the two study groups. This reduces the risk of confounding. However, in a study such as this, dealing with the global issue of antibiotic resistance and treatment plans, it is difficult to establish statistical correlations free of confounding factors, starting with the hurdle of exploring the extent of these confounders. There are a large number of identifiable and unidentifiable variables that exert a direct and indirect influence. Especially from an economic point of view, it is difficult to make a reliable statement about the sustainability of possible cost savings.

In conclusion, within the limitations of this retrospective study, our findings suggest that single-shot antibiotic prophylaxis may be a feasible approach in selected bone-related maxillofacial procedures such as orthognathic surgery, trauma, and augmentation and may reduce patient length of stay and total antibiotic consumption. However, special surgical techniques and hospital-specific aftercare may require adaptation of strict single-shot regimens. Therefore, maxillofacial departments should carefully assess their own patients. The implementation of a new in house-guideline with reduced antibiotic prophylaxis led to a significant reduction in the antibiotic use and might ad to the prevention of antibiotic-resistant infections. While our results support a reduction of prolonged prophylaxis in these settings, the overall evidence level remains low, and prospective studies are warranted to confirm these findings. In order to mitigate the emergence of multi-drug resistant pathogens, larger regional and even global studies will be needed. These will depend on local circumstances, health status, pathogen spectra and many other factors, some of which are still unknown.

## Data Availability

The datasets generated and/or analyzed during the current study are available from the corresponding author upon reasonable request.

## Declarations

### Study registration

The study was registered in the German Clinical Trials Registry (Registration ID: DRKS00029280).

## References

[1] Murray CJL, Ikuta K, Shunji AD, Hay, Simon I, Stergachis, Andy, Moore CE, Dolecek, Christiane, Naghavi M (2022) Global burden of bacterial antimicrobial resistance in 2019: a systematic analysis. The Lancet. 10.1016/S0140-6736(21)02724-0

[2] Layer F, Strommenger, Birgit, Cuny C, Werne G (2021) Eigenschaften, Häufigkeit und verbreitung von MRSA in Deutschland - Zur situation 2019/2020. Epidemiologisches Bull 40. 10.25646/9007

[3] Septimus EJ (2018) Antimicrobial resistance: an antimicrobial/diagnostic stewardship and infection prevention approach. Med Clin North Am 102:819–829. 10.1016/j.mcna.2018.04.005 30126573 10.1016/j.mcna.2018.04.005

[4] Hausmann J-E, Machtens, Egbert R, Eufinger Jürgen, Harald Kübler, Schliephake A, Henning (2012) Mund-, Kiefer- und gesichtschirurgie: operationslehre und -atlas. Springer-Verlag GmbH, Berlin, Heidelberg

[5] Ferri J, Druelle C, Schlund M, Bricout N, Nicot R (2019) Complications in orthognathic surgery: A retrospective study of 5025 cases. Int Orthod 17:789–798. 10.1016/j.ortho.2019.08.016 31495753 10.1016/j.ortho.2019.08.016

[6] Holtmann H, Hackenberg, Berit W, Bastian, Handschel Jörg (2020) BASICS- Mund-, Kiefer- und Plastische gesichtschirurgie. Elsevier GmbH Deutschland, München

[7] Gujer AK, Jacobsen C, Grätz KW (2013) Facharztwissen Mund-, Kiefer- und Gesichtschirurgie. Springer Verlag, Heidelberg

[8] (2022) Breakpoint tables for interpretation of MICs and zone diameters. European committee on antimicrobial susceptibility testing. Accessed Date 2022 https://www.eucast.org/fileadmin/src/media/PDFs/EUCAST_files/Breakpoint_tables/v_12.0_Breakpoint_Tables.pdf

[9] Resistenztestung und Antibiotika-Dosierung NAK- Nationales Antibiotika-Sensitivitätstest-Komitee. Accessed Date 2022 https://www.nak-deutschland.org/tl_files/nak-deutschland/NAK%202021/Dosierungen_NAK-20210105.pdf

[10] Habib AM, Wong AD, Schreiner GC, Satti KF, Riblet NB, Johnson HA, Ossoff JP (2019) Postoperative prophylactic antibiotics for facial fractures: a systematic review and meta-analysis. Laryngoscope 129:82–95. 10.1002/lary.27210 29756330 10.1002/lary.27210

[11] Oppelaar MC, Zijtveld C, Kuipers S, Ten Oever J, Honings J, Weijs W, Wertheim HFL (2019) Evaluation of prolonged vs short courses of antibiotic prophylaxis following Ear, Nose, Throat, and oral and maxillofacial surgery: A systematic review and Meta-analysis. JAMA Otolaryngol Head Neck Surg 145:610–616. 10.1001/jamaoto.2019.0879 31070697 10.1001/jamaoto.2019.0879 PMC6512286

[12] Mims C, Dockrell HM, Goering RV, Roitt, Ivan, Wakelin, Derek, Zuckermann M (2006) Medizinische Mikrobiologie- infektiologie: Mit virolgie und immunologie. Urban & Fischer Verlag, Elsevier GmbH, München

[13] Loke HY, Kyaw WM, Chen MIC, Lim JW, Ang B, Chow A (2019) Length of stay and odds of MRSA acquisition: a dose-response relationship? Epidemiol Infect 147:e223. 10.1017/S0950268819001110 31364542 10.1017/S0950268819001110 PMC6625199

[14] Liu JY, Dickter JK (2020) Nosocomial infections: a history of hospital-acquired infections. Gastrointest Endosc Clin N Am 30:637–652. 10.1016/j.giec.2020.06.001 32891222 10.1016/j.giec.2020.06.001

[15] Faul F, Erdfelder E, Lang AG, Buchner A (2007) G*Power 3: a flexible statistical power analysis program for the social, behavioral, and biomedical sciences. Behav Res Methods 39:175–191. 10.3758/bf03193146 17695343 10.3758/bf03193146

[16] Andreasen JO, Jensen SS, Schwartz O, Hillerup Y (2006) A systematic review of prophylactic antibiotics in the surgical treatment of maxillofacial fractures. J Oral Maxillofac Surg 64:1664–1668. 10.1016/j.joms.2006.02.032 17052593 10.1016/j.joms.2006.02.032

[17] (DGHM)DGfHuMeV (2024) S3-Leitlinie Perioperative und Periinterventionelle Antibiotikaprophylaxe. AWMF online. Accessed date AWMF-Nr. 029/022 https://register.awmf.org/de/leitlinien/detail/067-009

[18] Blatt S, Al-Nawas B (2019) A systematic review of latest evidence for antibiotic prophylaxis and therapy in oral and maxillofacial surgery. Infection. Springer Verlag GmbH. 10.1007/s15010-019-01303-8 10.1007/s15010-019-01303-8 30945142

[19] Garner MR, Sethuraman SA, Schade MA, Boateng H (2020) Antibiotic prophylaxis in open fractures: evidence, evolving issues, and recommendations. J Am Acad Orthop Surg 28:309–315. 10.5435/JAAOS-D-18-00193 31851021 10.5435/JAAOS-D-18-00193

[20] Domingo F, Dale E, Gao C, Groves C, Stanley D, Maxwell RA, Waldrop JL (2016) A single-center retrospective review of postoperative infectious complications in the surgical management of mandibular fractures: postoperative antibiotics add no benefit. J Trauma Acute Care Surg 81:1109–1114. 10.1097/TA.0000000000001232 27537516 10.1097/TA.0000000000001232

[21] Milic T, Raidoo P, Gebauer D (2021) Antibiotic prophylaxis in oral and maxillofacial surgery: a systematic review. Br J Oral Maxillofac Surg 59:633–642. 10.1016/j.bjoms.2020.09.020 34016464 10.1016/j.bjoms.2020.09.020

[22] Dammling C, Abramowicz S, Kinard B (2022) Current concepts in prophylactic antibiotics in oral and maxillofacial surgery. Oral Maxillofac Surg Clin North Am 34:157–167. 10.1016/j.coms.2021.08.015 34802615 10.1016/j.coms.2021.08.015

[23] Shridharani SM, Berli J, Manson PN, Tufaro AP, Rodriguez ED (2015) The role of postoperative antibiotics in mandible fractures: a systematic review of the literature. Ann Plast Surg 75:353–357. 10.1097/SAP.0000000000000135 24691320 10.1097/SAP.0000000000000135

[24] Zein Eddine SB, Cooper-Johnson K, Ericksen F, Brookes CC, Peppard WJ, Revolinski SL, Carver TW (2020) Antibiotic duration and outcome complications for surgical site infection prevention in traumatic mandible fracture. J Surg Res 247:524–529. 10.1016/j.jss.2019.09.050 31668431 10.1016/j.jss.2019.09.050

[25] Lovato C, Wagner JD (2009) Infection rates following perioperative prophylactic antibiotics versus postoperative extended regimen prophylactic antibiotics in surgical management of mandibular fractures. J Oral Maxillofac Surg 67:827–832. 10.1016/j.joms.2008.06.093 19304041 10.1016/j.joms.2008.06.093

[26] Bianchi T, Wolcott RD, Peghetti A, Leaper D, Cutting K, Polignano R, Rosa Rita Z, Moscatelli A, Greco A, Romanelli M, Pancani S, Bellingeri A, Ruggeri V, Postacchini L, Tedesco S, Manfredi L, Camerlingo M, Rowan S, Gabrielli A, Pomponio G (2016) Recommendations for the management of biofilm: a consensus document. J Wound Care 25:305–317. 10.12968/jowc.2016.25.6.305 27286663 10.12968/jowc.2016.25.6.305

[27] Fay A, Nallasamy N, Allen RC, Bernardini FP, Bilyk JR, Cockerham K, Cruz AA, Devoto M, Dolman PJ, Dutton JJ, Jordan DR, Kersten R, Kim YD, Lucarelli MJ, McNab AA, Mombaerts I, Mourits M, Nerad J, Perry JD, Rose G, Saeed P, Seah LL, Selva D, Sivak-Callcott J, Strianese D, Verity DH, Orbital S (2020) Perioperative prophylactic antibiotics in 1,250 orbital surgeries. Ophthalmic Plast Reconstr Surg 36:385–389. 10.1097/IOP.0000000000001565 31917766 10.1097/IOP.0000000000001565

[28] Ariyan SM, Lal J, Cheng A, Borah D, Chung GL, Conly KC, Havlik J, Lee R, Andrew WP, McGrath MH, Pribaz J, Young V, Leroy (2015) Antibiotic prophylaxis for preventing surgical-site infection in plastic surgery. Plast Reconstr Surg J 135:1723–1739. 10.1097/prs.0000000000001265 10.1097/PRS.0000000000001265 25724064

[29] Bartella AK, Lemmen S, Burnic A, Kloss-Brandstatter A, Kamal M, Breisach T, Holzle F, Lethaus B (2018) Influence of a strictly perioperative antibiotic prophylaxis vs a prolonged postoperative prophylaxis on surgical site infections in maxillofacial surgery. Infection 46:225–230. 10.1007/s15010-017-1110-4 29250713 10.1007/s15010-017-1110-4

[30] Posnick JC, Choi E, Chavda A (2016) Operative time, airway management, need for blood transfusions, and in-hospital stay for Bimaxillary, Intranasal, and Osseous Genioplasty surgery: current clinical practices. J Oral Maxillofac Surg 74:590–600. 10.1016/j.joms.2015.07.026 26303951 10.1016/j.joms.2015.07.026

[31] Barrier A, Breton P, Girard R, Dubost J, Bouletreau P (2009) [Surgical site infections in orthognathic surgery and risk factors associated]. Rev Stomatol Chir Maxillofac 110:127–134. 10.1016/j.stomax.2009.02.003 19410270 10.1016/j.stomax.2009.02.003

[32] Naros A, Naros CH, Awad D, Krimmel M, Kluba S (2023) Antibiotic prophylaxis and surgical site infections in orthognathic surgery - a retrospective analysis. BMC Oral Health 23:688. 10.1186/s12903-023-03391-3 37743500 10.1186/s12903-023-03391-3 PMC10518949

[33] Van Camp P, Verstraete L, Van Loon B, Scheerlinck J, Nout E (2021) Antibiotics in orthognathic surgery: a retrospective analysis and identification of risk factors for postoperative infection. Int J Oral Maxillofac Surg 50:643–648. 10.1016/j.ijom.2020.09.024 33162297 10.1016/j.ijom.2020.09.024

[34] Morris DE, Lo LJ, Margulis A (2007) Pitfalls in orthognathic surgery: avoidance and management of complications. Clin Plast Surg 34:e17–29. 10.1016/j.cps.2007.05.011 17692692 10.1016/j.cps.2007.05.011

[35] Patel PK, Morris DE, Gassman A (2007) Complications of orthognathic surgery. J Craniofac Surg 18:975 – 85; Quiz 986-8. 10.1097/scs.0b013e318068442c 17667699 10.1097/scs.0b013e318068442c

[36] Frieri M, Kumar K, Boutin A (2017) Antibiotic resistance. J Infect Public Health 10:369–378. 10.1016/j.jiph.2016.08.007 27616769 10.1016/j.jiph.2016.08.007

[37] Infektionen NRS (2017) Definitionen nosokomialer infektionen für die surveillance Im Krankenhaus-Infektions- surveillance-system (KISS-Definitionen). Robert Koch-Institut, Nordufer 20, Berlin

[38] Sorensen LT (2012) Wound healing and infection in surgery. The clinical impact of smoking and smoking cessation: a systematic review and meta-analysis. Arch Surg 147:373–383. 10.1001/archsurg.2012.5 22508785 10.1001/archsurg.2012.5

[39] Toyoda Y, Fu RH, Li L, Otterburn DM, Rohde CH (2018) Smoking as an independent risk factor for postoperative complications in plastic surgical procedures: a propensity score-matched analysis of 36,454 patients from the NSQIP database from 2005 to 2014. Plast Reconstr Surg 141:226–236. 10.1097/PRS.0000000000003963 29280887 10.1097/PRS.0000000000003963

[40] Schäfer C (2021) Häufigkeit und Verteilungsmuster von Frakturen im Mund-, Kiefer- und Gesichtschirurgischen Fachgebiet mit besonderem Hinblick auf sport - eine retrospektive Studie. doi: urn:nbn:de:bvb:29-opus4-149425

[41] Wienecke A, Kraywinkel K (2019) Epidemiologie von Kopf-Hals-Tumoren in Deutschland. Onkologe 25:190–200. 10.1007/s00761-019-0534-0

[42] Unbekannt (2021) Krebs in Mundhöhle und Rachen. Robert Koch-Institut. Accessed date 2022 https://www.krebsdaten.de/Krebs/DE/Content/Krebsarten/Mundhoehle_Rachenkrebs/mundhoehle_rachen_node.html

